# Effect of Oblique-Angle Sputtered ITO Electrode in MAPbI_3_ Perovskite Solar Cell Structures

**DOI:** 10.1186/s11671-017-2326-z

**Published:** 2017-10-03

**Authors:** Kun-Yi Lee, Lung-Chien Chen, Yu-June Wu

**Affiliations:** 1 Department of Electrical Engineering, China University of Science and Technology, No. 245, Sec. 3, Academia Road, Taipei, 115 Taiwan; 2 Department of Electro-optical Engineering, National Taipei University of Technology, 1, 3 Sec., Chung-Hsiao E. Rd, Taipei, 106 Taiwan; 3 Graduate Institute of Optomechatronics Engineering, China University of Science and Technology, No. 245, Sec. 3, Academia Road, Taipei, 115 Taiwan

**Keywords:** Oblique-angle sputtered ITO, MAPbI_3_ perovskite, Wettability

## Abstract

This investigation reports on the characteristics of MAPbI_3_ perovskite films on obliquely sputtered ITO/glass substrates that are fabricated with various sputtering times and sputtering angles. The grain size of a MAPbI_3_ perovskite film increases with the oblique sputtering angle of ITO thin films from 0° to 80°, indicating that the surface properties of the ITO affect the wettability of the PEDOT:PSS thin film and thereby dominates the number of perovskite nucleation sites. The optimal power conversion efficiency (Eff) is achieved 11.3% in a cell with an oblique ITO layer that was prepared using a sputtering angle of 30° for a sputtering time of 15 min.

## Background

Indium tin oxide (ITO) is a transparent conductive material that comprises indium oxide (In_2_O_3_) and tin oxide (SnO_2_). It is widely used in liquid crystal displays, light-emitting diodes, and solar cells owing to its visible transparency of approximately 96*%* and conductivity of around 10 Ω/sq [1–5]. Several methods for improving the resistance and the transmittance of ITO films have been studied, including annealing and sputtering with various gas ratios and operating pressures [5–8]. The optoelectronic properties of obliquely sputtered ITO films have been reported upon [9, 10]. As an ITO film is deposited, it grows as a film with a tilted columnar structure at an angle on a substrate, owing to the shadow effect. The columnar ITO film exhibits a different morphology, anisotropic optical properties, and anisotropic resistivity [10].

Recently, solar cells with perovskite materials, such as CH_3_NH_3_PbI_3_, as an active layer have received much interest owing to their favorable power conversion efficiencies [11–18]. Most perovskite solar cells have transparent conductive oxide (TCO) glass, such as ITO or FTO (fluorine-doped tin oxide), as the substrate. However, the optoelectronic properties of an isotropic TCO film differ from those of an anisotropic TCO film. Therefore, this work develops planar perovskite solar cells using CH_3_NH_3_PbI_3_ (MAPbI_3_) perovskites on oblique ITO substrates that are prepared glancing angle deposition (GLAD). This investigation examines the optical, structural, and surface properties of MAPbI_3_ perovskite films on oblique ITO substrates that have been annealed at various temperatures and sputtered for various times. The relationships between the performance of the perovskite solar cell and the properties of the perovskite films are discussed.

## Methods

**Fig. 1:**
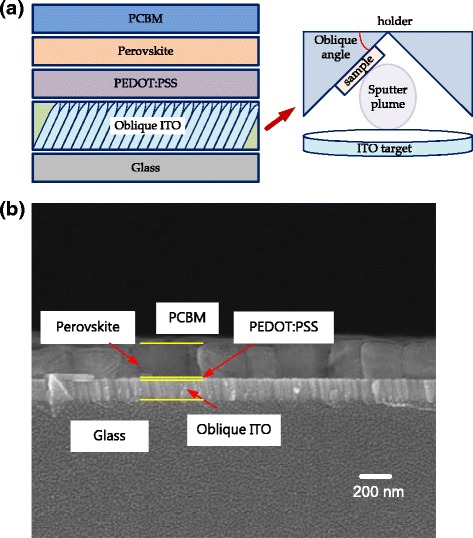
**a** Schematic cross section of completed structure and oblique sputtering system. **b** Cross-sectional FESEM image of sample with 30°-tilted sputtered oblique ITO

In this investigation, ITO glass was cut into small pieces of size 1.5 × 1.5 cm^2^ be used as substrates. The ITO glass substrates were thoroughly cleaned using acetone, ethanol, and deionized (DI) water in an ultrasonic oscillator for 5 min and dried with nitrogen. An ITO film was deposited onto the ITO glass substrate by sputtering at various oblique angles using ITO targets, as presented in Fig. 1a. The working gas and pressure were pure argon and 5 mTorr, respectively. After deposition, the films were annealed at 300 °C for 30 min.

Glass substrates that were coated with the obliquely sputtered ITO films were used for the perovskite solar cells. PEDOT:PSS films were prepared by spin-coating the oblique ITO glass substrates at 5000 rpm for 30 s. After spin-coating, the film was annealed at 110 °C for 10 min. The perovskite layer was deposited using two-step spin-coating onto the PEDOT:PSS/oblique ITO glass substrate at 1000 rpm for 10 s and 5000 rpm for 20 s. During the step at 5000 rpm for 20 s, the wet spinning film was quenched by dropping 100 μl of anhydrous toluene onto it. The perovskite precursor solutions were prepared using 1.25 mmol methylammonium bromide and 1.25 mmol PbI_2_ (with a purity of 99.999%) that was dissolved in 1 mL cosolvent. The volume ratio of dimethyl sulfoxide (DMSO) to γ-butyrolactone (GBL) was 1:1. After spin-coating, the film was annealed at 100 °C for 10 min. Then [6,6]-phenyl-C_61_-butyric acid methyl ester (PCBM) was dissolved in chlorobenzene (20 mg/mL) and spin-coated on perovskite layers at 3000 rpm for 30 s, forming a 50-nm-thick film as an electron transport layer. Finally, an Ag electrode with a thickness of 20 nm was deposited by thermal evaporation to complete the structure of the device. The sample was covered with a shadow mask that defined an active area of 0.5 cm × 0.2 cm during the deposition. Figure 1a schematically depicts the complete structure. Figure 1b shows the cross-sectional FESEM image of the sample with the 30°-tilted obliquely sputtered ITO.

## Results and Discussion

The crystalline microstructures of the films were observed using an X-ray diffractometer. A field-emission scanning electron microscope (FESEM) was used to observe the surface morphology of the samples. The current density–voltage (*J*–*V*) characteristics of the solar cells were measured using a Keithley 2420 programmable source meter under irradiation by a 1000 W xenon lamp. The irradiation power density on the surface of the cell was calibrated to 1000 W/m^2^.

**Fig. 2:**
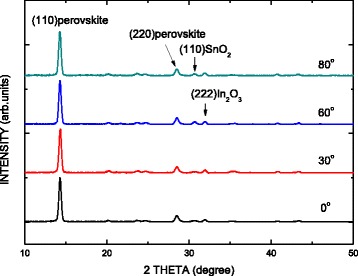
XRD patterns of MAPbI_3_ perovskite films on PEDOT:PSS/oblique ITO layer/glass for various oblique angles

Figure 2 shows the XRD patterns of the MAPbI_3_ perovskite films on PEDOT:PSS/oblique ITO layer/glass at various oblique angles. The four mean peaks at 14.28°, 28.5°, 30.61°, and 31.93° correspond to the (110) perovskite, (220) perovskite, (110) SnO_2_, and (222) In_2_O_3_ planes, respectively. As the sputtering angle increases from 0° to 60°, the (110) SnO_2_ is formed by the incorporation of Sn atoms. The size of the crystal domain can be calculated using Scherrer’s equation [19]. The sizes of the crystal domains of the MAPbI_3_ perovskite films in the samples are approximately 71.8 nm. Therefore, the sizes of the crystal domains of the MAPbI_3_ perovskite are not affected by the oblique ITO layer.

**Fig. 3:**
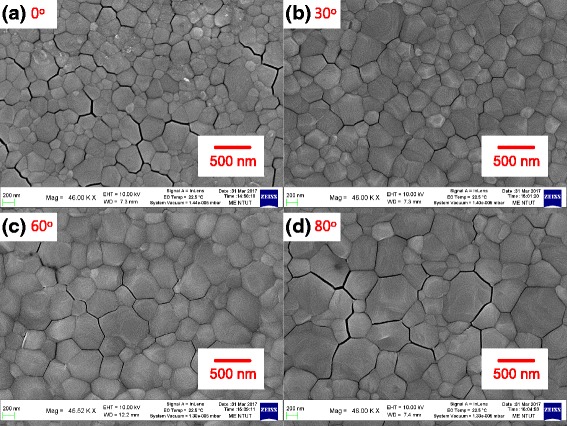
**a**–**d** SEM images of MAPbI_3_ perovskite films on PEDOT:PSS/oblique ITO layer/glass for various oblique angles

**Fig. 4:**
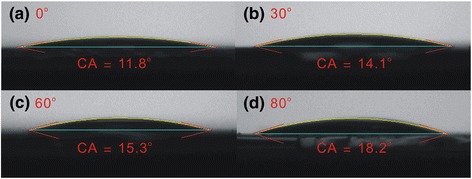
**a**–**d** Images that show contact angle of water on PEDOT:PSS/oblique ITO layer/glass for various oblique angles. CA contact angle

**Fig. 5:**
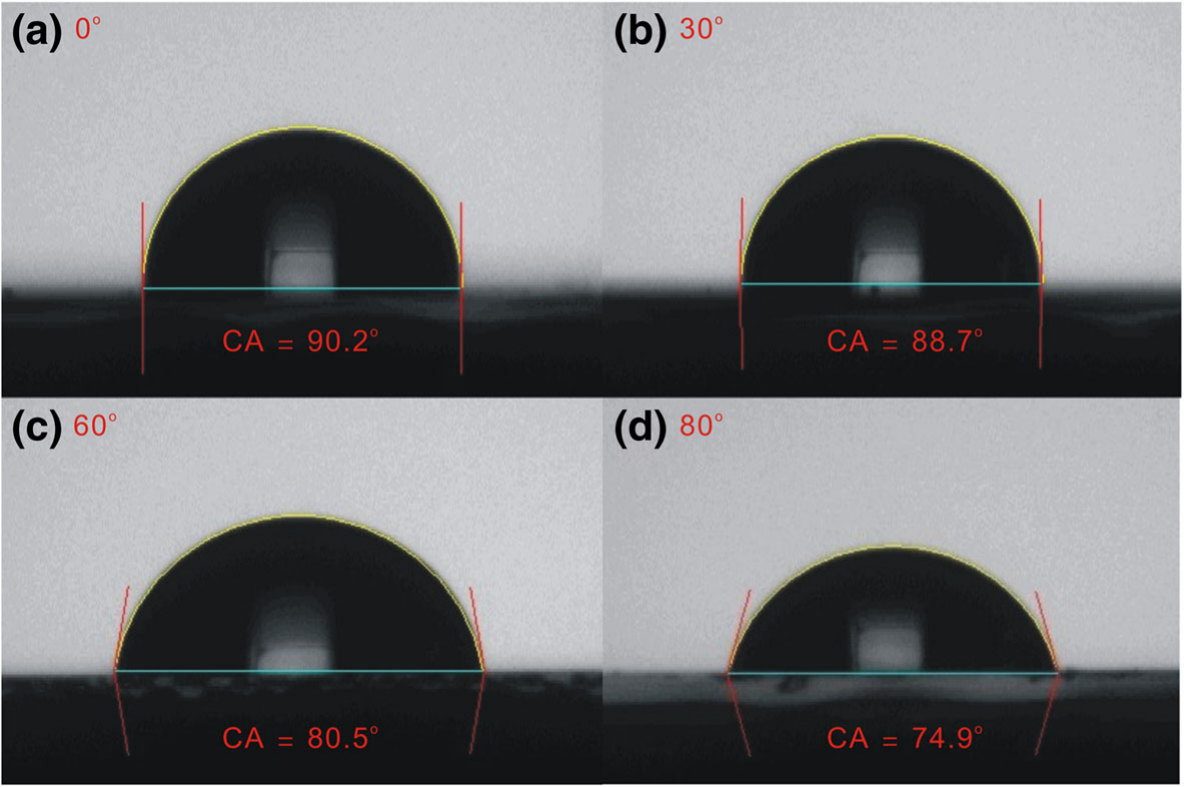
**a**–**d** Images that show contact angle of water on oblique ITO layer/glass for various oblique angles

Figure 3 shows SEM images of MAPbI_3_ perovskite films on an oblique ITO layer/glass for various oblique angles. The grain (or particle) size of the MAPbI_3_ perovskite films increases with the oblique sputtering angle from 0° to 80°, revealing that the surface properties of the ITO influence the number of perovskite nucleation sites. Since the ITO is not in direct contact with the perovskite thin film and a PEDOT:PSS thin film is inserted between the ITO and the perovskite, the surface properties of the ITO should not directly influence the properties of the perovskite thin films. Hence, the wettability of the PEDOT:PSS thin films [20] is related to the surface properties of the ITO. Therefore, the different grain sizes in the MAPbI_3_ perovskite films may be related to the wettability of the substrate [21, 22]. Experiments on the contact angle of a water droplet were carried out to assess the wettability of the PEDOT:PSS thin films on the different ITO/glass samples, as shown in Fig. 4. The contact angle is proportional to the size of the grains in the MAPbI_3_ thin film, indicating that the nucleation and crystal growth of a MAPbI_3_ thin film can be controlled by varying the surface wettability of the PEDOT:PSS/oblique ITO/glass. Contact angle images of the oblique ITO/glass samples were obtained to understand the variation of the surface wettability of the PEDOT:PSS/oblique ITO/glass samples, as depicted in Fig. 5. The wettability of the PEDOT:PSS/oblique ITO/glass samples is inversely proportional to the wettability of the oblique ITO/glass samples, so the vertical distributions of the hydrophilic PSS polymers and hydrophobic PEDOT polymers can be manipulated by varying the surface wettability of the oblique ITO/glass sample. PSS polymers are suggested to be distributed mostly in the upper surface of the PEDOT:PSS thin film when the substrate has a hydrophobic surface (Fig. 5a), resulting in a small water droplet contact angle on the PEDOT:PSS thin film (Fig. 4a). The experimental results (XRD and SEM) demonstrate that the MAPbI_3_ grains are multi-crystalline MAPbI_3_ particles [23].

**Fig. 6:**
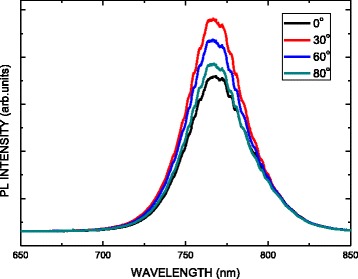
PL spectra of MAPbI_3_ perovskite films on PEDOT:PSS/oblique ITO layer/glass for various oblique angles

Figure 6 presents the photoluminescence (PL) spectra of the MAPbI_3_ perovskite films on PEDOT:PSS/oblique ITO/glass for various oblique angles. One main peak is observed at 768 nm, corresponding to emission by MAPbI_3_. The finding is supported by the XRD results. The PL emission energy of the MAPbI_3_ perovskite is not affected from beneath the oblique ITO layer. Additionally, the different PL intensities of the MAPbI3 films on ITOs sputtered with various oblique angles were obtained as a result of the separation of the light-induced exciton. A better interface between PEDOT:PSS and perovskite provided better exciton separation, inducing a stronger PL quenching effect. Therefore, ITO at an oblique angle of 80° exhibited the best exciton separation from the perovskite layer to PEDOT:PSS, owing to the favorable surface wettability of the PEDOT:PSS/oblique ITO, as shown in Fig. 4.

**Fig. 7:**
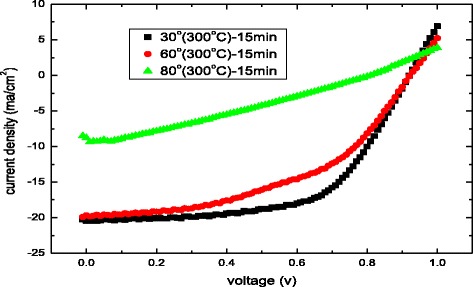
Current density–voltage (*J*–*V*) of solar cells based on MAPbI_3_ perovskite with oblique ITO layer sputtered at various oblique angles

Figure 7 plots the current density–voltage (*J*–*V*) curve of the solar cells that are based on MAPbI_3_ perovskite with an oblique ITO layer that is sputtered at various oblique angles and undergoes heat treatment at an annealing temperature of 300 °C. The sputtering time is 15 min. Table 1 presents the resulting power conversion efficiency (Eff), short-circuit current density (*J*_sc_), open-circuit voltage (*V*_oc_), and fill factor (FF) of the MAPbI_3_ solar cells. The performance of the device degrades as the sputtering angle of the oblique ITO layer increases, because the oxygen content in the oblique ITO layers and their resistance increase with the sputtering angle [10]. Maximum efficiency can be achieved following deposition at an oblique angle of 30° owing to the favorable conductivity.

**Table 1:** Parameters of perovskite MAPbI_3_ film on oblique ITO layer with various oblique angles

Oblique angle (°)	*V*_oc_ (V)	*J*_sc_ (mA/cm^2^)	FF (%)	Eff (%)
30	0.92	20.46	60.00	11.30
60	0.922	19.68	49.11	8.91
80	0.80	8.75	31.06	2.19

**Fig. 8:**
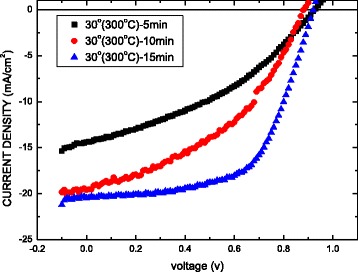
Current density–voltage (*J*–*V*) of solar cells based on MAPbI_3_ perovskite with oblique ITO layer sputtered for various sputtering times

Figure 8 plots the current density–voltage (*J*–*V*) curves of the solar cells that are based on MAPbI_3_ perovskite with the oblique ITO layer sputtered for various sputtering times, before undergoing heat treatment at an annealing temperature of 300 °C. Table 2 presents the corresponding power conversion efficiency (Eff), short-circuit current density (*J*_sc_), open-circuit voltage (*V*_oc_), and fill factor (FF) of the MAPbI_3_ solar cells. Optimum efficiency is reached when the sputtering time of the oblique ITO layer is 15 min because of the thickness of the layer and its good conductivity. The best device is obtained using this deposition angle, with *J*_SC_ = 20.46 mA/cm^2^, *V*_OC_ = 0.92 V, FF = 60.00%, and Eff = 11.30%.

**Table 2:** Parameters of perovskite MAPbI_3_ film on oblique ITO with various sputtering times

Sputtering time (min)	*V*_oc_ (V)	*J*_sc_ (mA/cm^2^)	FF (%)	Eff (%)
5	0.92	14.40	37.78	5.042
10	0.88	19.60	42.59	7.359
15	0.92	20.46	60.00	11.30

## Conclusions

In summary, this work demonstrated the characteristics of MAPbI_3_ perovskite films on the PEDOT:PSS/oblique-sputtered ITO/glass substrates that were fabricated using various sputtering times and sputtering angles. The device performance was optimized using an oblique ITO layer that was prepared by sputtering at 30° for 15 min, with a short-circuit current density (*J*_SC_) = 20.46 mA/cm^2^, open-circuit voltage (*V*_OC_) = 0.92 V, fill factor (FF) = 66.0%, and power conversion efficiency (Eff) = 11.3%. The performance of the device degrades as the sputtering angle of the oblique ITO layer increases from 30° to 80° because the resistance of the device increases with the sputtering angle. Although oblique ITO layers improve the scattering of incident light, the high resistivity degrades the performance of the device. Therefore, optimum efficiency can be achieved by deposition at an oblique angle of 30° owing to the conductivity.

## Abbreviations

FESEM: Field-emission scanning electron microscope
GLAD: Glancing angle deposition
ITO: Indium tin oxide
*J*–*V*: Current density–voltage
MAPbI_3_: CH_3_NH_3_PbI_3_
PEDOT:PSS: Poly(3,4-ethylenedioxythiophene) polystyrene sulfonate
TCO: Transparent conductive oxide
XRD: X-ray diffractometer
